# Advancing personalized medicine in brain cancer: exploring the role of mRNA vaccines

**DOI:** 10.1186/s12967-023-04724-0

**Published:** 2023-11-18

**Authors:** Feng Lin, Emma Z. Lin, Misa Anekoji, Thomas E. Ichim, Joyce Hu, Francesco M. Marincola, Lawrence D. Jones, Santosh Kesari, Shashaanka Ashili

**Affiliations:** 1 CureScience Institute, 5820 Oberlin Drive Ste 202, San Diego, CA 92121 USA; 2 University of California San Diego, La Jolla, CA 92093 USA; 3 Therapeutic Solutions International, Oceanside, CA 92056 USA; 4 Sonata Therapeutics, Watertown, MA 02472 USA; 5 Saint John’s Cancer Institute, Santa Monica, CA 90404 USA

**Keywords:** mRNA vaccine, Personalized medicine, Brain cancer, Tumor antigen, Combinatorial therapies, Adjuvant, Checkpoint inhibitors

## Abstract

Advancing personalized medicine in brain cancer relies on innovative strategies, with mRNA vaccines emerging as a promising avenue. While the initial use of mRNA vaccines was in oncology, their stunning success in COVID-19 resulted in widespread attention, both positive and negative. Regardless of politically biased opinions, which relate more to the antigenic source than form of delivery, we feel it is important to objectively review this modality as relates to brain cancer. This class of vaccines trigger robust immune responses through MHC-I and MHC-II pathways, in both prophylactic and therapeutic settings. The mRNA platform offers advantages of rapid development, high potency, cost-effectiveness, and safety. This review provides an overview of mRNA vaccine delivery technologies, tumor antigen identification, combination therapies, and recent therapeutic outcomes, with a particular focus on brain cancer. Combinatorial approaches are vital to maximizing mRNA cancer vaccine efficacy, with ongoing clinical trials exploring combinations with adjuvants and checkpoint inhibitors and even adoptive cell therapy. Efficient delivery, neoantigen identification, preclinical studies, and clinical trial results are highlighted, underscoring mRNA vaccines' potential in advancing personalized medicine for brain cancer. Synergistic combinatorial therapies play a crucial role, emphasizing the need for continued research and collaboration in this area.

## Introduction

Personalized medicine aims to revolutionize healthcare by providing tailored treatments based on an individual’s unique characteristics. Genetic information of the host and target plays a crucial role in determining disease susceptibility and treatment response [1, 2]. By utilizing genomic analysis, biomarker identification, risk assessment, tailored treatment strategies, and continuous monitoring, personalized medicine seeks to improve patient outcomes while minimizing adverse effects and reducing healthcare costs. In the field of brain cancer, personalized medicine holds great promise due to the complexity and heterogeneity of tumors, as well as individual variations in treatment response.

Brain cancer poses a significant threat to health, with approximately 1% of annual cancer diagnoses in the United States attributed to this condition. The rapid growth and invasion of malignant tumors in the brain or nearby structures can disrupt vital brain functions. Treatment options for brain cancer, including surgery, radiation therapy, and chemotherapy, vary in effectiveness depending on tumor type, location, and stage. However, challenges such as the difficulty of accessing and removing tumors in critical brain regions without damaging healthy tissue [3], and the presence of blood–brain barrier (BBB) limit the efficacy of current treatment approaches. Furthermore, brain tumors often exhibit heterogeneity, with different cell populations having varying sensitivities to therapies. These factors necessitate personalized treatment strategies tailored to each patient’s unique circumstances.

Immunotherapy has emerged as a promising approach for brain cancer treatment, harnessing the body's innate and adaptive defenses to seek and destroy transformed cells. Immune checkpoint inhibitors, such as pembrolizumab and nivolumab, have shown some success in treating certain types of brain cancer [4, 5] by blocking proteins that inhibit immune responses, thereby enhancing the ability of immune cells to recognize and attack cancer cells. Other immunotherapeutic approaches, including tumor and biomarker-based vaccines [6], monoclonal antibody drugs [7], and chimeric antigen receptor (CAR) T cell therapies are also being investigated [8, 9]. These immunotherapies aim to directly target cancer biomarkers, induce tumor regression, and modulate the suppressive tumor microenvironment, all with the goal of improving overall survival rates. However, several challenges hinder the effectiveness of immunotherapy for brain cancer. The presence of the BBB restricts immune cell access to the tumor site, potentially diminishing treatment efficacy [10]. Additionally, the immunosuppressive brain microenvironment and tumor heterogeneity further complicate the activation of immune response [11]. A deeper understanding of the intricate interactions between the immune system and brain cancer cells is crucial for the development of more effective and personalized treatment options.

Cancer vaccines, which educate the immune system about cancer cells to enable their recognition and elimination, represent an attractive form of immunotherapy. Prophylactic cancer vaccines against hepatitis B (HBV)-related liver cancer and human papilloma virus (HPV)-related cervical cancer and head and neck cancer have already been approved by the U.S. FDA [12]. Therapeutic cancer vaccine have been extensively studied in both preclinical and clinical settings, with Bacillus Calmette-Guerin (BCG) and Sipuleucel-T (Provenge) being antigen nonspecific and antigen specific FDA-approved examples. Recent studies, including a phase 3 clinical trial of a dendritic cell vaccine have reported success at prolonging survival in glioblastoma (GBM) patients [13]. Chen et al. developed a novel cancer cell-based therapy by modifying tumor cells to release immunomodulatory agents and induce cancer cell apoptosis. The engineered therapeutic tumor cells (ThTCs) effectively eliminated glioblastoma tumors and promoted antitumor immune responses in mice, offering a potential immunotherapy for glioblastoma tumor and other solid tumors [14]. Other type of vaccines such as personalized or shared neoantigen peptide vaccines for brain cancer have been obtaining promising results [15]. Advances in single-cell and RNA sequencing technologies have facilitated the identification of cancer biomarkers, allowing for the development of personalized neoantigen vaccines for brain cancer. These vaccines target specific neoantigens recognized by T cells, offering a highly immunogenic approach for personalized vaccination [16]. Overall, targeting either tumor cells or benign cells within the cancer cellular network should aim to enhance, either directly or indirectly, immunogenic cell death. This is likely the primary driver that transforms a poorly immunogenic tumor into an immune active, immune infiltrated phenotype that is more responsive to immunotherapy [17, 18].

To induce specific immune responses again tumor cells, therapeutic vaccines must activate broad immune responses, particularly specific cytotoxic T cell response. Genetic vaccines, including DNA and RNA vaccines, have the ability to prime both T and B cell responses, activating cellular and humoral immune responses and inducing an adaptive immune response to the encoded target antigen [19]. An example of this are studies demonstrating DNA-based therapeutic vaccines targeting HPV exhibit impressive efficacy in phase II clinical trials and are progressing to phase III [20, 21]. Compared to DNA vaccines, mRNA vaccines are believed to have a better safety profile and a more adaptable production process [22, 23]. The recent approvals of mRNA vaccines for COVID-19 have further highlighted their potential of mRNA vaccine. Notably, COVID-19 mRNA vaccination induced significant antibody [24, 25] and memory B cell responses against full-length SARS-CoV2 spike protein and the spike receptor binding domain (RBD) [25, 26], along with robust T cell response [27, 28]. Encouraging results from preclinical studies and clinical trials with mRNA cancer vaccines and mRNA infectious disease vaccines have inspired biotechnological and pharmaceutical industries to focus on this promising area of research [29, 30].

This review aims to explore the factors, combinations, and optimizations necessary to maximize the efficacy of mRNA cancer vaccines and delivery systems. It will also discuss the clinical applications of mRNA vaccines in advancing personalized medicine for brain cancer. Additionally, the review will consider future considerations for transitioning mRNA vaccines from the laboratory to the clinic. By shedding light on the role of mRNA vaccines in brain cancer treatment, we aim to contribute to the growing body of knowledge and provide valuable insight into the potential of mRNA-based immunotherapies for improving brain cancer treatment outcomes.

### mRNA vaccine delivery technology, route, and adjuvant exploration

mRNA is intrinsically not stable, but over the past decades, several technological innovations have enabled mRNA to be a more favorable vaccine platform. Developments in modification of mRNA backbone and untranslated regions by codon optimization as well as innovative selection of internal ribosome entry sites have resulted in creation of molecules with increased stability and RNase resistance, as well as augmented translation efficiency [31, 32]. Introduction of efficient non-viral in vivo delivery of mRNA has been achieved by formulating mRNA into lipid nanoparticles (LNPs) using peptides, cationic nano-emulsions, lipids, and polymers [31, 33, 34], and the technology has been reviewed by Hou et al. and others [35–38]. Alternatively, viral like particles may provide the specificity that maybe lacking with LNPs [39, 40].

Ongoing improvements and optimizations to the technology are underway. Given that the dendritic cell (DC) resides at the center of the immunological universe, being the only cell capable of activating naïve T cells, one unique approach involves specifically targeting mRNA to these cells. Zhang et al. developed a minimalist nanovaccine with C1 LNPs that could efficiently deliver mRNA into dendritic cells with simultaneous Toll-like receptor 4 (TLR4) activation and induced robust T cell activation [41]. In a different variation on the theme, Verbeke et al. [42] developed mRNA Galsomes, a nanoparticle platform co-delivering mRNA, glycolipid antigen, and α-GC to antigen-presenting cells after intravenous administration. In mice, mRNA Galsomes triggered a robust tumor-specific immune response. Tumor growth decreased, achieving complete rejection in 40% of animals. Combining mRNA Galsomes with a PD-L1 checkpoint inhibitor reduced tumor outgrowth and increased survival, highlighting their potential in cancer immunotherapy. Fornaguera et al. created poly(beta aminoesters) polymer nanoparticles as efficient mRNA delivery systems, with a formulation protocol for disease prevention and cure using mRNA vaccines [43]. Fan YN et al. investigated cationic lipid-assisted nanoparticles (CLAN) for mRNA vaccine delivery. CLAN effectively stimulated dendritic cell maturation and activated antigen-specific T cells in vitro and in vivo. Intravenous immunization with CLAN containing mRNA encoding ovalbumin resulted in a robust OVA-specific T-cell response and inhibited tumor growth in an E·G7-OVA lymphoma model [44]. Similarly, Mai et al. [45] used positively charged protamine to concentrate mRNA and form a stable polycation-mRNA complex. Encapsulating this complex with cationic liposomes (LPC) resulted in enhanced dendritic cell maturation and potent anti-tumor immune response in vitro and in vivo.

These targeted delivery systems have been utilized via alternative rounds of administration for example, intranasal immunization of mice with cationic LPC containing mRNA encoding cytokeratin 19 resulted in a robust cellular immune response and inhibited tumor growth in a Lewis lung cancer model. Persano et al. developed a lipopolyplex mRNA vaccine comprising a poly-(β-amino ester) polymer mRNA core enclosed within an EDOPC/DOPE/DSPE-PEG lipid shell. This vaccine enters dendritic cells, stimulates interferon-β and interleukin-12 expression through Toll-like receptor 7/8 signaling. In a mouse tumor model with B16-OVA lung metastatic tumors, treatment with the lipopolyplex mRNA led to over 90% reduction in tumor nodules [46]. Haabeth et al. developed charge-altering releasable transporters (CARTs) for efficient mRNA vaccine delivery. CARTs target antigen presenting cells (APCs) in lymphoid organs via intravenous injection and local APCs via subcutaneous injection in a mouse model. Co-formulating CARTs with mRNA and Toll-like receptor ligands enables transfection and activation of target cells, leading to an effective immune response capable of treating and curing mice with large, established tumors [47]. Overall, these studies appear to be a “second generation” in the development of mRNA therapeutics in that they are targeting specific immune cells. Further understanding of dendritic cell subsets will allow for additional improvements in efficacy of this approach.

Dendritic cells naturally respond to recognition of immunological adjuvants by enhancement of antigen presenting activity. Accordingly, administration of various adjuvants may increase efficacy of mRNA activity through increasing dendritic cell number and activity. Hess et al. found genetic adjuvant can boost immunogenicity of mRNA vaccine. Co-delivering GM-CSF mRNA with tumor-associated antigen mRNA significantly enhances the cytotoxic T cell response in animal tumor models [48]. Li et al. developed CpG2018B, activating immune responses through TLR9. CpG2018B and an mRNA-based neoantigen cancer vaccine were encapsulated in lipid nanoparticles (LNPs) and intratumorally administered to melanoma mouse models. Combing CpG and the mRNA vaccine enhanced the antitumor effect [49]. A novel vaccine design exploits its dual role in adaptive and innate immunity. Combing free and protamine-complexed mRNA, it induces balanced adaptive immune responses with antigen-specific CD4 + and CD8 + T cells, as well as sustains memory responses. In a mouse tumor model, the two-component mRNA vaccine demonstrates robust antitumor activity against OVA-expressing tumor cells, serving as both preventive and therapeutic intervention [50].

Leveraging of various nanotechnologies has also been used to augment mRNA immune stimulatory activity. For example, a novel adjuvant-pulsed mRNA vaccine nanoparticle has been developed, containing ovalbumin-coded mRNA and a palmitic acid-modified TLR7/8 agonist (C16-R848). The lipid-PEG-coated NP with C16-R848 adjuvant significantly enhances OVA-specific CD8 + T cells expansion and infiltration into the tumor sites. In mice models, this approach effectively induces anti-tumor immunity against OVA-expressing lymphoma and prostate cancer. Administered before tumor engraftment, the vaccine prevents tumor growth by 84% compared to the control, while post-engraftment administration results in a 60% reduction in tumor growth [51].Van Lint et al. formulated a TriMix mRNA adjuvant vaccine that encodes CD70, CD40 ligand, and a constitutively active Toll-like receptor 4. Intra-tumoral delivery stimulated antitumor T-cell responses in animal models, resulting in systemic therapeutic antitumor immunity [52]. Bialkowski et al. demonstrated that intra-lymphatic E7-TriMix mRNA vaccine induced CD8 ( +) T lymphocyte migration into the tumor and controlled tumor growth effectively. Combining mRNA immunization with cisplatin treatment led to complete regression of clinically relevant tumors [53].

The recent advancements have elevated the field of mRNA therapeutics from just a novel method of antigen delivery to a practical treatment modality with practical ability to modulate immunity to tumors which conventionally have been seen as poorly immunogenic. With the tools now available to break antigen-specific tolerance found in cancers, the selection of appropriate antigens has become paramount.

### mRNA vaccine for cancer

mRNA is a single-stranded macromolecule transcribed from DNA in the cell nuclei. Once transcribed, this endogenous mRNA is read by ribosomes to translate the genetic information into proteins in the cytoplasm [54]. Leveraging this natural process, scientists have synthesized mRNA for vaccines. The mRNA vaccine has been utilized in cancer treatment to prompt cells to produce cancer-specific proteins/neoantigens [55]. These neoantigens can induce an immune response against cancer cells, making mRNA vaccine a promising approach in cancer immunotherapy. It has rapidly become recognized that mRNA vaccines possess multiple advantages compared to traditional approaches [55–57]. Firstly, mRNA vaccines can be customized to target specific tumor antigens or neoantigens unique to each patient's cancer cells through a process that involves tumor removal, sequencing to identify these unique antigens, and then developing a personalized vaccine. While promising, this approach can be prolonged and costly, with biotech estimates placing the cost per patient at around 350,000 USD. Alternatively, there are mRNA strategies that prompt immunogenic cell death, directly releasing tumor antigens from the cancer cells. These methods not only expedite the process but also allow for the creation of tailored vaccines as a subsequent step after tumor extraction. The personalized nature of mRNA vaccines facilitates a precisely target immune response against cancer cells, ensuring minimal impact on healthy cells. While the development and manufacturing of these vaccines are swift, enabling rapid adjustment to ever-changing tumor traits and mutations, there’s a caveat: tumor sequencing and epitope definition for each patient might introduce delays. Still, the versatility of mRNA vaccine is evident in their compatibility with other cancer therapies. When paired with treatments like chemotherapy or checkpoint inhibitors, they can significantly amplify the efficacy. This synergy with established treatments could enhance overall therapeutic outcomes. Moreover, by eliciting both cellular and humoral immune defenses, mRNA vaccines may offer a robust, long-term shield against cancer cells, potentially diminishing the odds of a recurrence.

The initial clinical utilization of mRNA vaccines was in oncology in which numerous clinical trials have been conducted to evaluate the safety and efficacy of mRNA vaccines in various types of cancer [58, 59]. Early clinical results have shown promising outcomes, with evidence of immune responses against tumor cells and some instances of tumor shrinkage. Findings from the phase IIb KEYNOTE-942 trial indicate that a personalized mRNA cancer vaccine, mRNA-4157/V940, combined with pembrolizumab, improved recurrence-free survival in high-risk melanoma patients regardless of tumor mutational burden. The vaccine encodes patient-specific tumor neoantigens, stimulating an immune response [59]. The approach shows promise for melanoma and potentially other cancers such as pancreatic cancer [60].

Research efforts are also focused on refining delivery methods, stability, and immunogenicity specifically for cancer applications. As mRNA vaccine technology advances and more data is gathered from clinical trials, it is expected that the potential of mRNA vaccines for cancer treatment will continue to be explored and optimized. The ability to customize vaccines based on the individual genetic profile of each patient's tumor offers a promising avenue for personalized cancer therapy. However, further research and larger-scale clinical studies are required to determine the full potential and integration of mRNA vaccines into standard cancer treatment protocols. Several challenges need to be addressed through ongoing research and development. These challenges include optimizing delivery systems, stabilizing mRNA molecules, and modulating immunogenicity to maximize the potential of mRNA vaccines in cancer immunotherapy.

### mRNA cancer vaccine clinical trials

Clinical trials exploring mRNA cancer vaccines are underway, transforming the standard of case in oncology treatment. These trials leverage mRNA technology to activate the immune system against cancer cells, offering personalized medicine for improved outcomes. With diverse vaccine options targeting different malignancies, researchers are diligently evaluating their efficacy and safety. These groundbreaking trials hold immense potential to transform the landscape of cancer therapy, offering hope for cancer patients. The related trials both completed and ongoing have been listed in Table 1.

**Table 1:** mRNA vaccine clinical trials for cancer

mRNA Vaccine	Adjuvant/intervention	Indication	Phase	Trial number	Sponsor	Reference
Personalized mRNA vaccine (Autogene Cevumeran)	Atezolizumab	Advanced tumors including pancreatic cancer	I	NCT03289962	Genentech, (collaborator: BioNTech)	[60]
	Atezolizumab, mFOLFIRINOX	Pancreatic cancer	II	NCT05968326		
Personalized mRNA vaccine	N/A	Esophageal cancer, NSCLC	I	NCT03908671	Stemirna Therapeutics	
	Sintilimab	Advanced solid tumor	I	NCT05949775		
Personalzed mRNA vaccine SW1115C3	N/A	Solid tumor	I	NCT05198752		
Personalized mRNA vaccine	N/A	Esophageal, gastric, pancreatic, and colon cancers	I	NCT03468244	Changhai Hospital, Shanghai	
Personalized mRNA vaccine	Sintilimab	Liver cancer	I	NCT05761717	Shanghai Zhongshan Hospital	
Personalized neoantigen mRNA vaccine	N/A	Solid tumor	I	NCT05359354	Peking Union Medical College Hospital	
EBV mRNA vaccine	N/A	EBV-positive advanced malignant tumors	I	NCT05714748	West China Hospital	
HBV mRNA vaccine	N/A	HBV-positive liver cancer	I	NCT05738447	Peking Union Medical College Hospital	
BNT113 (HPV16 E6/E7 mRNA- LPX vaccine)	Pembrolizumab	HPV16 + _head and neck cancer, PDL1 postive	II	NCT04534205	BioNTech SE	
Neoantigen mRNA vaccine ABOR2014(IPM511)	NA	Liver cancer	I	NCT05981066	Peking Union Medical College Hospital	
mRNA neoantigen tumor vaccine	Combination with PD-1/L1	Gastric cancer, esophageal cancer, liver cancer	I	NCT05192460	NeoCure	
DCs loaded with mRNA from tumor tissue, hTERT and Survivin	N/A	Prostate cancer	I/II	NCT01197625	Oslo university hospital	
CV9103 mRNA		Prostate cancer	I/II	NCT00831467	CureVac	[65]
BNT112 containing mRNA targeting 5 antigens expressed in prostate cancder (RNA-LPX)	Cemiplimab	Prostate cancer	I	NCT04382898	BioNTech SE	
Tumor mRNA-lipid particles(LPs)	N/A	Lung cancer	I	NCT05660408	University of Florida	
mRNA vaccine BI1361849 (CV9202)	Anti-PDL1 Durvalumab Anti-CTLA3 Tremelimumab	NSCLC Results posted on Clinicaltrials.gov	I/II	NCT03164772	Ludwig Institute for cancer research	
V941(mRNA-5671/V941)	Pembrolizumab	NSCLC, colorectal cancer or pancreatic cancer	I/II	NCT03948763 Completed	Merck Sharp & Dohme LLC	
mRNA-4157	Pembrolizumab	Solid tumor	I	NCT03313778	ModernaTX, Inc	
WT1 mRNA loaded DCs	Combined with Chemotherapy	Malignant pleural mesothelioma	I/II	NCT02649829	University Hospital, Antwerp	
TAA-loaded DCs	N/A	Colorectal cancer	I/II	NCT01885702	Radboud University	
CEA mRNA and peptide loaded DCs (electroporated)		Colorectal cancer	I	NCT00228189	Radbound Universtity Medical Cener	[71]
Personalized primary tumor mRNA loaded DOTAP liposome vaccine	Anti-PD1	Melanoma	I	NCT05264974	University of Florida	
mRNA-4157	Pembrolizumab	Melanoma	II	NCT03897881	Moderna TX, Inc	[74]
DCs-loaded with TAA mRNA	N/A	Melanoma	I	NCT01456104	Memorial Sloan Kettering Cancer Center	
TriMix-DC (mRNA-loaded DCs)		Melanoma	I	NCT01066390	Universitair Ziekenhuis Brussel	[66, 67]
Tumor mRNA transfected DCs	IL-2	Melanoma	I/II	NCT01278940	Olso University Hospital	[70]
mRNA encoding Melan-A, MAGE-A1, MAGE-A3, Survivin, GP100 and Tyrosinase	GM-CSF s.c	Melanoma	I/II	NCT00204607	University Hospital Tuebingen	[63]
NCI-4650 mRNA vaccine (personalized)		Melanoma or epithelial cancer	I/II	NCT03480152	NCI	[16]
CT7, MAGE-A3 and WT1 mRNA-electroporated Langerhans cells (LCs)		Multiple myeloma	I	NCT01995708	Memorial Sloan Kettering Cancer Center	[73]
WT1 mRNA-DCs	N/A	AML	II	NCT01686334	Zwi Berneman	
WT1 mRNA -DCs	N/A	Myelodysplastic syndromes, AML	I/II	NCT03083054	University of Campinas, Brazil	
WT1 mRNA-DCs		AML	I/II	NCT00834002	University Hospital, Antwerp	[72]
GRNVAC1 mRNA encoding hTERT-LAMP1 -DCs		AML	II	NCT00510133	Asterias Biotherapeutics, Inc	
mRNA plus lysate-loaded DCs	Pegylated-interferon alpha-2A and Filgrastim	Cutaneous Angiosarcoma	I	NCT05799612	MD Anderson Cancer Center	
CMV-DC (CMV pp65-LAMP mRNA-loaded DC)	Tetanus toxoid	GBM	I/II	NCT00639639	Duke University	
DCs transfected with mRNA from tumor stem cells, surviving and hTERT	Adjuvant temozolomide	GBM	II/III	NCT03548571	Oslo University Hospital	
CMV pp65-LAMP mRNA-pulsed autologous DC vaccine	N/A	GBM	I	NCT02366728	Duke University	
WT1 mRNA-loaded DC vaccine	Temozolomide	Glioma	I/II	NCT04911621	University Hospital, Antwerp	
Personalized primary tumor mRNA and full length of pp65-LAMP (RNA-LPs)	N/A	pHGG and GBM	I	NCT04573140	University of Florida	
CV09050101 mRNA	N/A	GBM or astrocytoma	I	NCT05938387	CureVac	
CMV-pp65-LAMP mRNA- loaded DCs	Varillumab and Temozolomide	GBM	II	NCT03688178	Duke University	
CMV pp65-LAMP mRNA-loaded DCs	CMV-ALT, GM-CSF	GBM	I/II	NCT00639639	Duke University	[76, 77]
CMV mRNA DCs	Nivolumab	GBM	I/II	NCT02529072	Duke University	
pp65-shLAMP mRNA-DCs	Td, and GM-CSF	GBM	II	NCT02465268	Immunomic Therapeutics, Inc	
WT1 mRNA-loaded DCs	Temozolomide	GBM	I/II	NCT02649582	Unviersity Hospital, Antwerp	
Tumor stem cell derived mRNA-transfected DCs	Combined with standard treatment	GBM	I/II	NCT00846456	Oslo University Hospital	[75]
Personalized mRNA-DCs		GBM	I/II	NCT02709616	Guangdong 999 Brain Hospital	[78]

The first mRNA vaccine clinical trial for cancer was conducted in renal cell carcinoma (RCC). The trial aimed to induce T-cell responses against multiple tumor-associated antigens (TAAs) to improve overall survival. Conducted between 2003 and 2005, investigators administered an intradermal mRNA-based vaccine encoding six TAAs (MUC1, CEA, Her2/neu, telomerase, survivin, and MAGE-A1) to 30 metastatic RCC patients. Results revealed that long-term survivors exhibited immunological responses to the antigens, establishing a clear link between survival and immune responses to TAAs encoded by the naked mRNA vaccine [61, 62]. In a phase I/II trial, Weide et al. [63] admonished protamine-stabilized mRNAs coding for Melan-A, Tyrosinase, gp100, Mage-A1, Mage-A3, and Survivin to 21 metastatic melanoma patients. Ten patients received keyhole limpet hemocyanin (KLH) with the vaccine, and GM-CSF was used as an adjuvant. KLH patients experienced decreased Foxp3 + /CD4 + regulatory T cells, while non-KLH patients had reduced myeloid suppressor cells (CD11b + HLA-DR lo monocytes). Two of four evaluated patients showed increased vaccine specific T cells. One of 7 patients with measurable disease showed a complete response.

Several similar studies were reviewed by Bidram et al. [64], highlighting the associated challenges. They emphasized the potential of combining mRNA vaccines with immune-checkpoint blockade to enhance cell-mediated immunity. Viability of this approach was observed in other tumors, for example, the mRNA vaccines CV9103 and CV9104 were developed for prostate cancer treatment [65]. They utilize RNActive® formulation by CureVac (Tubingen, Germany), enabling optimized translational activity and immune stimulation. CV9103, the precursor to CV9104, targets four prostate-specific antigens: PSA, PSMA, PSCA, and STEAP. Phase I and II trials of CV9103 demonstrated safe administration, high immunogenicity rates, and favorable clinical outcomes, including prolonged stabilization of PSA levels. CV9104, with additional mRNA molecules encoding PAP and MUC1, showed antigen-specific immune responses in 5 out of 6 patients, eliciting immune changes in CD4 + and CD8 + T cells [65].

Utilizing mRNA in DC-based cancer vaccines shows promise due to DCs role in linking innate and adaptive immune responses. Van Nuffel et al. loaded the mRNAs of gp100, MAGE-A3, and MAGE- C2 into the DCs via the pST1-SIINFEKL vector, resulting in significant levels of functional CD4 + and CD8 + cells in melanoma patients [66, 67]. Since the peptides from loaded mRNAs must be coupled with the MHC compound to mount the anti-tumoral immune responses, the chimeric lysosome-associated membrane protein-1 (LAMP-1) with the tumoral antigen can facilitate this process [68]. Similarly, Su et al. have shown that the mRNA of tumoral antigen/LAMP-1 can enhance the peptide/MHC-I presentation and the anti-tumoral immune responses in affected patients [69]. However, Kyte et al. [70] vaccinated melanoma patients with autologous tumor-mRNA loaded DCs in a phase I/II trial. The study showed the DC-vaccine was safe, feasible, and induced in vivo T-cell response against transfected tumor-mRNA antigens. Lesterhuis et al. [71] compared CEA mRNA-DC with CEA peptide loaded DC vaccine in 16 colorectal cancer patients. CEA peptide-specific T cells were detected in 8 of 11 patients in the peptide group, but none in the mRNA group, possibly due to different antigen processing pathways or T-cell repertoires. Van de Velde et al. [72] reported a non-randomized single-center study for acute myeloid leukemia in 50 patients. The study demonstrated better survival in patients treated with allogeneic hematopoietic stem cell transplantation (HCT) and Wilms’ tumor protein (WT1) mRNA transfected DCs vaccine compared to control groups. Chung et al. [73] conducted a phase 1 vaccine trial using autologous Langerhans-type dendritic cells (LCs) electroporated with CT7, MAGE-A3, and Wilms tumor 1 (WT1) mRNA in multiple myeloma patients after autologous stem cell transplantation. The study demonstrated the vaccine's safety and ability to induce antigen-specific immune reactivity.

Therapeutic cancer vaccinations have traditionally focused on differentiation antigens, cancer-testis antigens, and overexpressed antigens. Recent studies have highlighted the recognition of neoantigens by T cells, providing a highly specific and immunogenic target for personalized vaccination. Cafri et al. developed a process utilizing tumor-infiltrating lymphocytes to identify immunogenic mutations in patients' tumors. These validated neoantigens and predicted neoepitopes were then combined into a single mRNA construct for vaccination in metastatic gastrointestinal cancer patients. Their Phase I/II trial demonstrated the safety of the vaccine and induced mutation-specific T cell responses against predicted neoepitopes not previously detected [16]. In a phase I trial, researchers used a personalized mRNA neoantigen vaccine, Autogene Cevumeran (a patient specific vaccine incorporating a maximum of 20 neoantigens per patient), in combination with atezolizumab and chemotherapy to treat pancreatic ductal adenocarcinoma (PDAC) patients. The vaccine induced neoantigen-specific T cells in 8 out of 16 patients and correlated with longer recurrence-free survival, independent of patients' immune fitness. This combination treatment shows promise in delaying PDAC recurrence [60], and is gearing up for a subsequent phase II trial. In a phase 2 trial, the combination of mRNA-4157 (V940) and pembrolizumab showed reduced risk of death or developing distant metastasis in high-risk melanoma patients compared to pembrolizumab alone. The combination induced high response rates and improved distant metastasis-free survival. The study demonstrated the potential of personalized mRNA vaccines in improving outcomes for melanoma patients [74].

Clinical trials with mRNA-based vaccines in brain cancer have shown to be promising. Vik-Mo et al. conducted a clinical study with cancer stem cell (CSCs)-mRNA loaded DCs vaccination in glioblastoma patients’ post-radio-chemotherapy. An immune response was identified in all seven patients, with no adverse autoimmune events. Progression-free survival was significantly longer in vaccinated patients compared to controls (Vik-Mo et al. [75]). Cytomegalovirus pp65 is expressed in > 90% of GBM specimens but not surrounding normal brain, it is an ideal tumor-specific target. Vaccination with of pp65 mRNA-pulsed DCs in patients with GBM can extend survival [76], especially when followed by dose-intensified (DI) TMZ and adjuvant GM-CSF. Despite increased Treg proportions, patients receiving pp65- DCs showed long-term progression-free survival and overall survival [77]. Zhu et al. [78] reported a GBM case treated with a combination immunotherapy of tumor-associated antigen (TAAs) and neoantigen mRNA DC vaccines, anti-PD-1, poly I:C, and cyclophosphamide integrated with standard chemoradiation therapy. The patient remained disease-free for 69 months with no immunotherapy-related adverse events, indicating the safety and feasibility of this integrated combination immunotherapy for long-term treatment in this patient.

In summary, an increasing number of mRNA cancer vaccine clinical trials are emerging. Our online search for studies relating to cancer and mRNA vaccines revealed over 80 clinical trials. Of these, 36 are currently active or under recruiting participants.

### mRNA cancer vaccine combination with checkpoint inhibitors and CAR-T cell therapy (preclinical)

A potentially groundbreaking approach in cancer treatment would combine mRNA-based vaccines and checkpoint inhibitors, revolutionizing personalized medicine. This strategy leverages the immune system to destroy cancer cells, offering unprecedented precision and effectiveness. The marriage of these therapies is reshaping oncology and inspiring hope in patients and researchers.

In a preclinical study, Wang et al. [79] engineered a cancer vaccine utilizing mRNA and immune checkpoint-blocking siRNA delivered through lipid-coated calcium phosphate nanoparticles. This innovative vaccine elicited robust immune responses, effectively impeding melanoma growth in mice. Simultaneous administration of PD-L1 siRNA and the mRNA vaccine synergistically amplified T cell activation, resulting in remarkable suppression of tumor growth and metastasis. In parallel, Liu et al. employed nanoparticles (NPs) for delivering an mRNA vaccine targeting the tumor antigen MUC1 in a triple-negative breast cancer (TNBC) 4T1 animal model. They combined the mRNA vaccine with an anti-CTLA-4 monoclonal antibody to augment the anti-tumor effects. The findings revealed that the NP-based mRNA vaccine successfully induced a potent and specific in vivo cytotoxic T lymphocyte response against TNBC 4T1 cells. Furthermore, the combination immunotherapy of the vaccine and anti-CTLA-4 monoclonal antibody exhibited a significantly enhanced anti-tumor immune response compared to either the vaccine or monoclonal antibody alone [80]. Chimeric antigen receptor (CAR) technologies have revolutionized the field of hematological malignancies, however, have had minimal success in solid tumors. One innovative approach to increase efficacy is boosting through vaccination. This process involves DCs recruitment, increased tumor antigen uptake, and priming of anti-tumor T cells. CAR-T-derived IFN-gamma plays a crucial role in promoting antigen spreading, enabling complete responses even with antigen-negative tumors. Vaccine boosting is a clinically viable strategy against solid tumors [81].

These advancements, when coupled with the principles of personalized medicine guided by various omics strategies, hold immense potential for revolutionizing glioma therapy. Furthermore, exploring novel immunotherapy approaches, particularly in the context of GBM, may hold the key to enhancing current immune checkpoint inhibitor therapies, considering the historical resistance of GBM to such interventions.

### The role of mRNA vaccines in brain cancer

The role of mRNA vaccines in brain cancer is an area of active research and development. Although mRNA vaccines have shown promise in other disease areas, their specific application for brain cancer is still in the early stages.

Due to their potency in stimulation of immunity, as well as the precedent of one FDA approved immunotherapy (Provenge), dendritic cells (DCs) have been a key focus in neoantigen and/or mRNA vaccine research for brain cancer. DC-based vaccines have been demonstrated to be effective to cancers including brain cancer glioma. A recent Phase 3 clinical study investigated the efficacy of adding a tumor lysate-based DC vaccine called DCVax-L to standard treatment for glioblastoma. The results indicated that adding DCVax-L extended the survival of patients with newly diagnosed and recurrent glioblastoma compared to those who received standard treatment alone. This suggests that DCVax-L could be a promising addition to current glioblastoma treatments [13]. A few studies of mRNA-based vaccines have been reported. The receptor for hyaluronan-mediated motility (RHAMM), an overexpressed antigen in brain tumors, has been recently identified as an immunogenic antigen through serological screening of cDNA expression libraries. Researchers investigated the therapeutic potential of DCs transfected with RHAMM mRNA in a glioma model. Mice treated with the vaccine showed significantly prolonged survival compared to control groups. Immunohistochemical analysis demonstrated an increased presence of CD4 + , CD8 + , and CD25 + activated T lymphocytes in the vaccinated group. These findings underscore the therapeutic potential of this approach in treating malignant glioma [82]. In a mouse glioma model, researchers explored the potential of DCs vaccination with IL13ra2 mRNA, a target for glioma immunotherapy. DCs were transfected with Il13ra2 mRNA using a constructed plasmid and injected into the glioma model. The group treated with DCs transfected with Il13ra2 mRNA exhibited a significant improvement in survival rate and increased numbers of CD4 + and CD8 + T lymphocytes. These results emphasize the potential of DC vaccination with Il13ra2 mRNA as a treatment approach for malignant glioma [83]. In a groundbreaking study, a DC vaccine with cancer stem cell (CSCs) mRNA was administered to seven patients with glioblastoma. Following radio-chemotherapy, the vaccine successfully induced an immune response in all patients, leading to a 2.9 times longer progression-free survival compared to controls. These promising results highlight the safety, tolerability, and potential of the vaccine to extend progression-free survival in glioblastoma patients [75]. A study evaluated the combination of dose-intensified temozolomide (DI-TMZ) and DCs pulsed with pp65 lysosome-associated membrane glycoprotein mRNA in glioblastoma patients. Following treatment, there was an increase in pp65 cellular responses, leading to long-term progression-free survival (PFS) and overall survival (OS) exceeding historical controls. Despite an increase in Treg proportions, the combination showed potential benefits in glioblastoma treatment [77].

The long-term durability and prevention of tumor relapse with mRNA vaccines in brain cancer remain under investigation. Nonetheless, ongoing research and advancements stemming from previous preclinical and clinical studies in mRNA vaccine technology hold promise.

### Novel tumor antigen identification in brain cancer for mRNA vaccine

To address the urgent need for effective treatments for brain cancer, particularly GBM, a highly lethal brain tumor despite existing treatment options, it is crucial to develop novel immune stimulatory interventions such as mRNA-based therapies. To achieve this, identifying new therapeutic targets or biomarkers is essential. Cell-surface proteins hold significant promise as targets due to their accessibility, involvement in critical signaling pathways, and dysregulation in cancer. Not only can they serve as potential targets for mRNA vaccine strategies, but they also have the potential to be utilized in chimeric antigen receptor (CAR) based immunotherapy approaches.

Brain tumors, including GBM, often exhibit molecular and genetic heterogeneity, meaning there are different subpopulations of cells within the tumor. Designing mRNA vaccines that can effectively target all tumor cell populations poses a challenge due to the diverse antigen profiles. Identifying the most appropriate antigens or neoantigens to target with mRNA vaccines requires thorough characterization of the patient's tumor. Obtaining accurate and comprehensive information about the specific antigens expressed by the tumor cells can be challenging, potentially limiting the efficacy of personalized mRNA vaccines.

Recent studies used bioinformatics analysis to identify potential neoantigens in brain cancer based on abnormal alternative splicing. Novel tumor antigens can be identified in certain cancer type of patients based on genomic, clinical, and mRNA sequence data from their normal and tumor tissues, the neoantigens have been listed in Table 2. In one study seeking to identify novel tumor antigens, Ye et al. analyzed 529 patient with low grade glioma (LGG) tumor samples and clinical data, and selected mutated, up-regulated, prognosis- and immune-related genes. They identified four potential tumor antigens restricted to LGG. These antigens, FCGBP, FLNC, TLR7, and CSF2RA were useful for development of mRNA cancer vaccines [84]. By using the same technology and analyses, Ye et al. further identified ARPC1B and HK3 as potential mRNA antigens for developing GBM mRNA vaccination, and the patients in IS2 were considered the most suitable population for vaccination in GBM [85]. PTBP1 and SLC39A1 were selected as antigens for LGG, and MMP9 and SLC16A3 were identified as antigens for GBM [86]. Zhou et al. identified KDR, COL1A2, and SAMD9 are potential antigens for developing mRNA vaccines against diffuse glioma [87]. TP53, IDH1, C3 and TCF12 four overexpressed and mutated tumor antigens were identified to be associated with poor prognosis and infiltration of antigen presenting cells in glioma patients. The four neoantigens are effective antigens for the development of anti-glioma mRNA vaccines. [88]. Zhong et al. analyzed over 1000 patients’ RNA-seq data and clinical information and identified four glioma antigens, namely ANXA5, FKBP10, MSN, and PYGL, to be associated with superior prognoses. They can also be used as potential antigens for anti-glioma mRNA vaccine production, specifically for patients in immune subtypes 2 and 3 [89]. GBM-associated surfaceome has been used to identify new therapeutic targets for GBM, and 11 specific potential targets found for potential immunotherapy such as mRNA strategy [90]. The immune subtypes of GBM were identified, and a potential neoantigen (LRP1) and patients suitable for vaccination were identified. These findings offer guidance for GBM immunotherapy development. Five potential tumor neoantigens (LRP1, TCF12, DERL3, WIPI2, and TSHZ3) were identified in GBM by selecting genes both with abnormal alternative splicing (upregulated) and gene frameshift mutations, in which LRP1 was significantly associated with APCs [91]. In another study, nine potential tumor antigens were identified, and four immune subtypes with distinct characteristics were validated. ADAMTSL4, COL6A1, CTSL, CYTH4, EGFLAM, LILRB2, MPZL2, SAA2, and LSP1 are candidate antigens, and IS1 and IS2 subtypes are suited for mRNA vaccination and immune checkpoint inhibitors, respectively. This study provides a framework for GBM immunotherapy strategies [92].

**Table 2:** Novel antigens identified in brain cancer suitable for mRNA vaccine

Brain cancer	Antigens	Reference
Low grade glioma (LGG)	FCGBP, FLNC, TLR7, CSF2RA	[84]
	PTBP1, SLC39A1	[86]
Diffuse glioma	KDR, COL1A2, SAMD9	[87]
GBM and glioma	ARPC1B, HK3	[85]
	MMP9, SLC16A3	[86]
	TP53, IDH1, C3, TCF12	[88]
	ANXA5, FKBP10, MSN, PYGL	[89]
	LRP1, TCF12, DERL3, WIPI2, TSHZ3	[90]
	ADAMTSL4, COL6A1, CTSL, CYTH4, EGFLAM, LILRB2, MPZL2, SAA2, and LSP1	[92]
	NAT1, FRRS1, GTF2H2C, BRCA2, GRAP, NR5A2, ABCB4, ZNF90, ERCC6L, ZNF813, and IS1/2A/2B	[93]
	ARHGAP9, ARHGAP30, CLEC7A, MAN2B1, ARPC1B and PLB1	[94]

Through the integration of RNA sequencing expression data and somatic mutation data from TCGA-GBM, TCGA-LGG, and CGGA datasets, ten biomarkers (NAT1, FRRS1, GTF2H2C, BRCA2, GRAP, NR5A2, ABCB4, ZNF90, ERCC6L, and ZNF813) have been identified as potential antigens suitable for glioma mRNA vaccine. Additionally, IS1/2A/2B have been determined to be suitable for mRNA vaccination [93]. Ultilizing various methods such as Gene Expression Profiling Interactive Analysis (GEPIA), cBioPortal, TIMER, RNA-seq data analysis, clustering analysis, graph learning-based dimensional reduction, and weighted gene co-expression network analysis (WGCNA), six overexpressed and mutated tumor antigens (ARHGAP9, ARHGAP30, CLEC7A, MAN2B1, ARPC1B and PLB1) have been identified as potential targets for developing anti-GBM mRNA vaccine. Furthermore, three immune subtypes of GBMs have been identified, with GBMs from the IS3 subtype showing a higher likelihood of benefiting from vaccination. The study also used WGCNA to identify potential vaccination biomarkers by clustering immune-related genes [94].

The comprehensive identification of tumor antigens in brain cancer, as detailed above, underscores the remarkable capabilities of contemporary "omics" technology. These advancements, harnessed for antigen discovery, not only bolster the potential of mRNA vaccine development for aggressive brain cancers but also pave the way for personalized, targeted immunotherapies. The integration of multifaceted analytical approaches has unearthed a wealth of potential targets, emphasizing the promising horizon of immunotherapeutic strategies for gliomas and other brain malignancies.

### Future directions

Combination therapies involving mRNA vaccines hold significant potential for enhancing the treatment of brain cancer. By combining mRNA vaccines with other treatment modalities, synergistic effects can be achieved to improve outcomes. Here are some potential combination strategies:

- Immune Checkpoint Inhibitors: Immune checkpoint inhibitors, such as antibodies targeting PD-1/PD-L1 or CTLA-4, have shown promise in enhancing immune responses against cancer cells. In tandem with mRNA vaccines, they can intensify the vaccine-induced immune response, overcoming immune suppression in the tumor microenvironment.
- Radiation Therapy: Radiation therapy is a standard treatment for brain cancer. Combining mRNA vaccines with radiation therapy can have a dual effect. Radiation therapy can induce tumor cell death, releasing antigens that can be targeted by the mRNA vaccine. Additionally, radiation therapy can also modulate the tumor microenvironment, potentially enhancing the effectiveness of the mRNA vaccine-induced immune response.
- Targeted Therapies: Targeted therapies, such as tyrosine kinase inhibitors or specific molecular inhibitors, are used to inhibit specific signaling pathways in cancer cells. Combining mRNA vaccines with targeted therapies can potentially enhance the vaccine-induced immune response while simultaneously targeting specific genetic alterations in the tumor cells.
- Chemotherapy: Chemotherapy drugs are commonly used in the treatment of brain cancer. Combining mRNA vaccines with chemotherapy can potentially increase the immune response against cancer cells while the chemotherapy targets rapidly dividing tumor cells.
- Adoptive Cell Therapy: Adoptive cell therapy involves the infusion of immune cells, such as CAR-T cells, that are genetically engineered to recognize and attack cancer cells. Combining mRNA vaccines with adoptive cell therapy can further enhance the anti-tumor immune response by priming the infused immune cells to recognize specific tumor antigens.

It is important to note that the choice and sequence of combination therapies should be carefully considered, considering the specific characteristics of the tumor and the patient's condition. As clinical trials progress, the focus remains on delineating the most efficacious combinational regimens. Here are some key areas that require further development:

- Biomarker Identification: Identifying and validating biomarkers crucial for predicting responses to mRNA vaccines and combination therapies can streamline patient selection, ensuring treatments are optimized for those with the highest likelihood of benefiting.
- Immunosuppressive Microenvironment: Given the brain tumor’s natural immunosuppressive nature, strategies to reverse or modulate this microenvironment will be key in enhancing mRNA vaccine effectiveness.
- Vaccine Design: Optimizing mRNA vaccine design, including the choice of antigens, adjuvants, and delivery systems, will be fundamental in boosting their potency against brain cancer.
- Combination Scheduling: Understanding the optimal scheduling and dosing of combination therapies is crucial. The timing of administering mRNA vaccines in relation to other treatments can influence their synergistic effects.
- Patient Monitoring and Holistic Care: Incorporating advanced imaging and diagnostic tools is essential for closely monitoring tumor responses and adjusting treatments, with real-time monitoring aiding in the early detection of potential side effects and complications. Furthermore, understanding the emotional and social challenges of brain cancer and giving all-around care, including mental support, is key to improving the patient's life.

The effective application of mRNA vaccines for brain cancer treatment demands a multifaceted approach, encompassing biomarker research, environmental modulation, optimized vaccine design, strategic combination scheduling, and a comprehensive, patient-centric care model.

## Conclusion

mRNA vaccines have the potential to revolutionize personalized medicine for brain cancer by targeting specific tumor antigens or neoantigens. They can stimulate a strong immune response while sparing healthy tissues. Benefits include durable immune responses, tumor-specific targeting, and adaptability for multiple antigens. Challenges include overcoming the blood–brain barrier, identifying optimal antigens, navigating the tumor microenvironment, and ensuring vaccine stability and scalability. Research and technological advancements are needed to refine delivery systems, understand tumor biology, optimize vaccine design, and develop effective combination therapies. Collaboration among researchers, clinicians, and industry partners is crucial for advancing personalized medicine in brain cancer using mRNA vaccines.

## Data Availability

Not available.
